# Long term relationship between farming damselfish, predators, competitors and benthic habitat on coral reefs of Moorea Island

**DOI:** 10.1038/s41598-021-94010-0

**Published:** 2021-07-15

**Authors:** William E. Feeney, Frédéric Bertucci, Emma Gairin, Gilles Siu, Viliame Waqalevu, Morgan Antoine, Thierry Lison de Loma, Serge Planes, René Galzin, David Lecchini

**Affiliations:** 1 Department of Behavioural Ecology and Evolutionary Genetics, Max Planck Institute for Ornithology, 82319 Seewiesen, Germany; 2 PSL Research University: EPHE-UPVD-CNRS, USR 3278 CRIOBE BP 1013, 98729 Papetoai, Moorea, French Polynesia; 3 Functional and Evolutionary Morphology Laboratory, University of Liege, Liege, Belgium; 4 Laboratoire de Biologie Des Organismes Et Ecosystèmes Aquatiques (BOREA), Université Des Antilles, MNHN, CNRS 8067, SU, IRD 207, UCN, Campus de Fouillole, 97159 Pointe-à-Pitre, Guadeloupe; 5 Laboratoire D’Excellence “CORAIL”, Perpignan, France; 6 Laboratory of Larval Rearing Management, United Graduate School of Agricultural Sciences, Kagoshima University, Kagoshima, 8908580 Japan; 7 School of Marine Studies, University of the South Pacific, 15387 Suva, Fiji

**Keywords:** Biodiversity, Community ecology, Population dynamics

## Abstract

Understanding the processes that shape biodiversity is essential for effective environmental management. Across the world’s coral reefs, algal farming damselfish (*Stegastes* sp.) modify the surrounding benthic community through their creation of algae “farms”. Using a long-term monitoring dataset (2005–2019) from Moorea Island, French Polynesia, we investigated whether the density of dusky damselfish (*Stegastes nigricans*) is associated with benthic habitat composition, the density of predators and/or competitors, and whether the survey area was inside or outside of a Marine Protected Area (MPA). We found no evidence that benthic cover or number of competitors were associated with dusky damselfish densities, both inside and outside MPAs. In contrast, fluctuations in dusky damselfish densities were negatively associated with the density of predators (e.g. Serranidae, Muraenidae and Scorpaenidae) in the preceding year in non-MPA areas, and both within and outside of MPAs when predator densities were high (2005–2010). These results suggest that healthy predator populations may be important for regulating the abundances of keystone species, such as algal farming damselfish, especially when predator densities are high.

## Introduction

Coral reefs are among the most biodiverse ecosystems on the planet, supporting roughly three million species and approximately 25% of all marine life. Unfortunately, the world’s coral reefs are under increasing pressure from local and global stressors (e.g. acidification, agricultural pesticides, plastics, temperature rise, and wastewater) that degrade reef condition and function^1–5^. These pressures risk causing drastic changes to coral reefs, such as “algal phase-shifts”, where scleractinian coral-dominated communities “shift” to less productive macroalgae- or algal turf-dominated communities^3,4,6,7^. Understanding the forces that affect community dynamics^8,9^ and identifying the processes that confer resilience to coral reefs in the face of change^10,11^, is thus a important challenge for marine ecologists.

Species interactions are important ecological processes that shape and regulate biodiversity. However, species do not affect surrounding biodiversity equally, and keystone species refer to those that disproportionately large effect compared to their abundance^12^. They influence ecosystem processes, such as by regulating prey populations (predators), regulating predator populations (prey), supporting other species through cooperative interactions (mutualists), linking mutualistic species (hosts, clients or partners), and by creating habitats that affect surrounding species (modifiers)^13^. Across the world’s coral reefs, some herbivorous fish are keystone species and the diverse array of species within this guild provides various ecosystem services, including those that may aid in the recovery of live hard coral^11,14–17^. By contrast, other keystone species, such as farming damselfish, can favor the development of algal turfs and defend them against other herbivorous fish^18–20^.

Damselfish of the genus *Stegastes* (Pomacentridae, herein ‘*Stegastes*’) are highly territorial species that develop dense turf algae patches (i.e. farms) which act as a food source^18,19^. Reliance on algae for sustenance varies across *Stegastes* species, spanning from facultative to obligate^21–23^. They actively control the algal species composition within their farms, and defend them against other herbivorous fish, invertebrate micro-herbivorous grazers and sea-urchins^17,19,20^. In order to provide substratum for their algal field, they can actively removed scleractinian corals, such as by biting the living tissue and cultivating dense algal lawns on the coral skeletons^24^. Correspondingly, the farms of several *Stegastes* species (e.g. *S. apicalis* and *S. nigricans*) can act as reservoirs of pathogens that cause coral diseases^25^. While these species can modify habitats around them, other species can regulate their density and distribution. For example, Precht et al.^24^ found that *Stegastes* densities were strongly regulated by predation risk, and Randazzo-Eisemann et al.^26^ found that predators are likely to play an important role in regulating the distribution of *Stegastes* and reducing the stress that they impose on the coral system. Overall, research conducted on farming damselfish over the last four decades has highlighted the influence of *Stegastes* population dynamics on the benthic cover of coral reefs and thus their potential usefulness as an ecological indicator, but also the potential effects some competitors and predators have on their density^18,19^.

While long-term studies have found that the density of *Stegastes-*associated turf can increase significantly as the damselfish take advantage of coral mortality^26–29^, few monitoring studies have tracked whether their abundance is associated with, and affected by, predator and competitor populations as well as benthic habitat composition^30^. Notably, Naim et al.^28^ showed that algal turf abundance increased significantly between 1993 and 2002 due to the expansion of *Stegastes* territories over time in some coral reefs at Reunion Island. During their monitoring across 64 Mesoamerican reefs, Randazzo-Eisemann et al.^26^ showed that the density of algal-gardening damselfish increased from 1.5 individuals per 100m^2^ in 2006 to 4.7 individuals per 100m^2^ in 2016, and that their density was strongly correlated with fleshy macroalgae cover. It is also recognised that some herbivorous fish and invertebrate micro-herbivorous grazers are competitors of farming damselfish by regulating the height of the algal canopy and inhibiting algal dominance on the reef^17,30^. However, sediment-rich algal turfs, that farming damselfish cultivate, may inhibit herbivory due to their high carbonate content as that may interfere with herbivorous fish digestion ^31^. Thus, a reduction in herbivory will favor first denser turf algae patches, and subsequently the farming damselfish population. Conversely, if predator populations are reduced due to overfishing, the farming damselfish population may increase^26^.

In the present study, we utilized an extensive long-term monitoring dataset (2005–2019) to investigate how the dusky gregory (*Stegastes nigricans*, Tahitian name: 'atoti) abundances, the abundance of predators and algal competitors, and benthic cover vary through time inside and outside Marine Protected Areas at Moorea Island (French Polynesia). Given this species’ close association with turf algae, we predicted that variations in the density of *S. nigricans* would correlate with variations in the proportion of living coral and algal covers. We also predicted a negative association between predators and/or competitors populations and *S. nigricans* density.

## Results

### Changes in *S. nigricans* density since 2005

*Stegastes nigricans* are common on the shallow reef flats around Moorea^32^. Thus, the shallow reef fish surveys from eight MPA and five non-MPA sites as part of the Fisheries Service of French Polynesia and the CRIOBE’s long-term monitoring efforts (2005–2019, Fig. 1)^33^ were used in this study (see *Methods* for more details.

**Figure 1:**
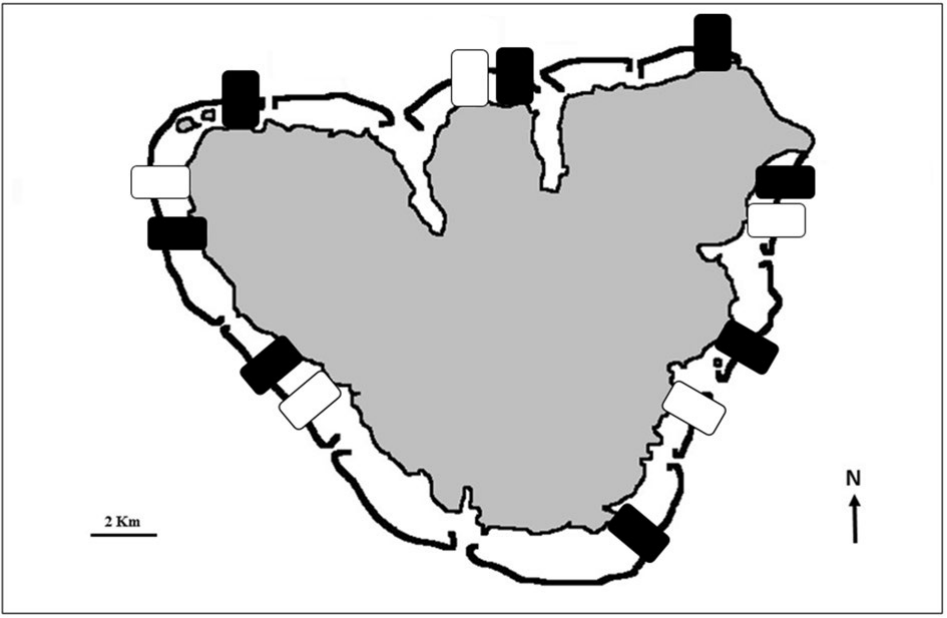
Map of Moorea Island, French Polynesia (drawn by the authors using PhotoFiltre 7 software [version 7.1.2—www.photofiltre.com]) and location of the 13 shallow reef flats (MPA sites—black rectangles; non-MPA sites—white rectangles) used in this study.

**Figure 2:**
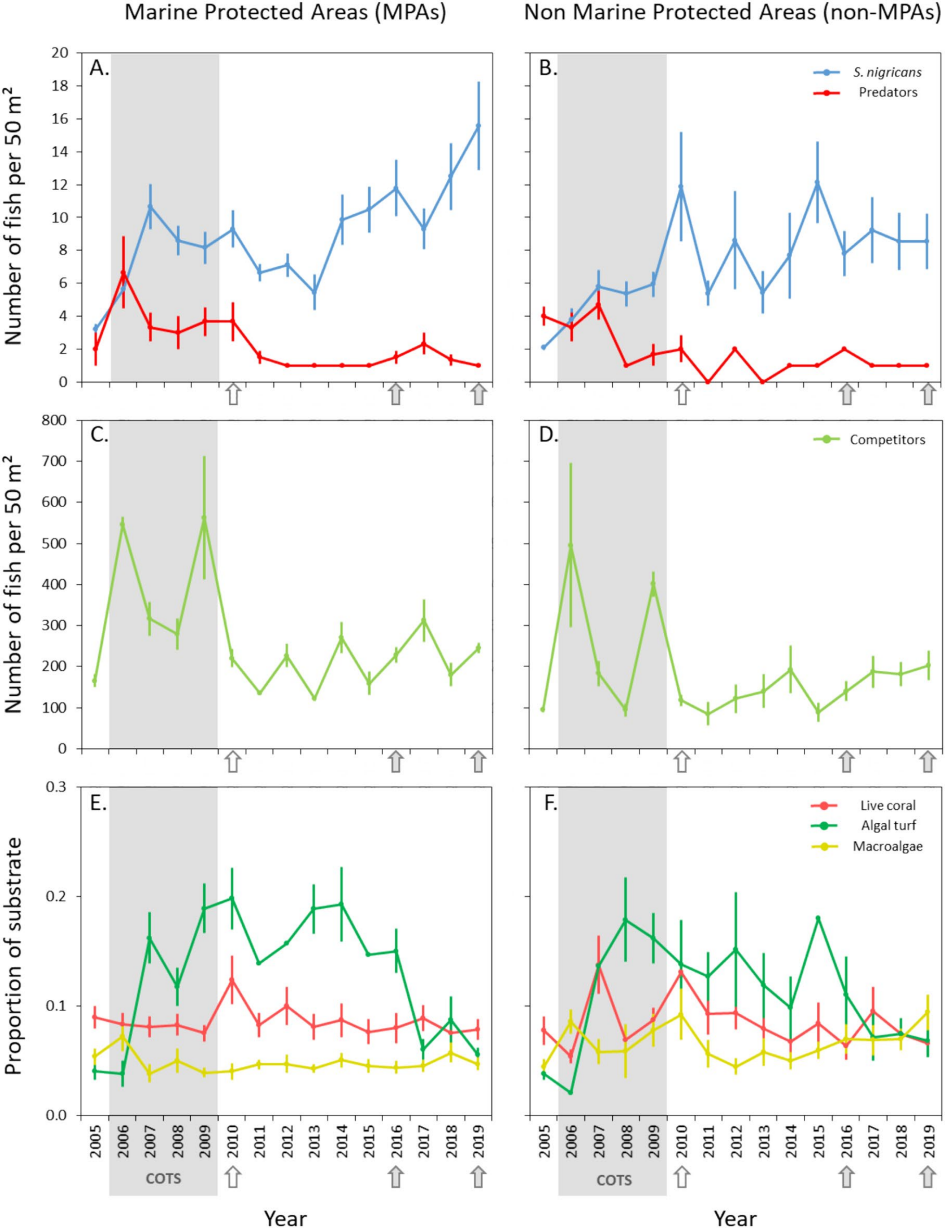
Number of fish per 50 m^2^ of *Stegastes nigricans* and predators (**A**, **B**), competitors (**C**, **D**) and proportions of live coral, algal turf, and macroalgae (**E**, **F**) from 2005 to 2019 at Moorea on reef flats within no-take marine protected areas (MPA) and on non-protected sites (non-MPA), where fishing is allowed. Values are mean ± S.E. A COTS outbreak is indicated in grey (2006–2009), the 2010 hollow arrow indicates a cyclonic event (Cyclone Oli), and the filled arrows indicate weak bleaching events on the reef flats in 2016 and 2019.

From 2005 to 2019, the density of *S. nigricans* increased significantly inside and outside MPAs (Mann–Kendall tests, τ14 = 0.52, *P* = 0.008) (Fig. 2A and B). In 2005, the density was 2.64 ± 0.25 fish per 50m^2^ (mean ± SE) on reef flats throughout all sites. In 2019, the density was 12.06 ± 2.19 fish per 50m^2^. A significant increase in fish density was observed both inside and outside of the MPAs from 2005 to 2010, before cyclone Oli hit Moorea. Then, a stabilization was observed between 2010 and 2013. Following this, the density of *S. nigricans* increased again, but only within MPA areas, until 2019 (Fig. 2A). Outside of the MPAs, the density of *S. nigricans* showed more variability and remained at the same level from 2016 onwards (Fig. 2B).

### Changes in fish predators and competitors since 2005

In 2005, the predator density inside the MPAs was 2.00 ± 1.00 fish per 50m^2^. Following this, there was an increase to 6.67 ± 2.19 fish per 50m^2^ in 2006, before stabilizing between 3.00 ± 1.00 and 3.67 ± 1.20 fish per 50m^2^ from 2007 to 2010. From 2011 to 2019, predator density was similar to 2005 levels, ranging between 1.00 ± 0.00 and 2.00 ± 0.82 fish per 50m^2^ (Fig. 2A). The density of predators inside the MPAs showed a significant decreasing trend (Mann–Kendall test, τ_14_ = -0.43, *P* = 0.008). The same pattern was observed outside the MPAs; however, this trend was not significant (Mann–Kendall test, τ14 = −0.36, *P* = 0.056). Taken together, while predator densities in 2005 and 2019 were both similar, the larger increase in recruitment within the MPA sites in 2006 appears responsible for the statistical decrease in abundance within the MPA versus the non-significant result outside of the MPA sites. For competitors, no significant trend was detected either inside or outside MPAs throughout the years (densities ranged from 110 to 260 fish per 50m^2^), except for two peaks in abundance in 2006 and 2009, with densities higher than 500 fish per 50m^2^ (MPAs: Mann–Kendall tests, τ_14_ = −0.16, *P* = 0.30; non-MPAs: Mann–Kendall test, τ_14_ = 0.12, *P* = 0.55) (Fig. 2C and D).

### Substrate cover

From 2005 to 2019, no significant trend was found for live hard corals (MPAs: Mann–Kendall tests, τ_14_ = −0.05, *P* = 0.86; non-MPAs: Mann–Kendall test, τ_14_ = −0.19, *P* = 0.36), algal turf (MPAs: Mann–Kendall tests, τ_14_ = −0.04, *P* = 0.88; non-MPAs: Mann–Kendall test, τ_14_ = −0.24, *P* = 0.29) and macroalgae (MPAs: Mann–Kendall tests, τ_14_ = −0.08, *P* = 0.74; non-MPAs: Mann–Kendall test, τ_14_ = 0.19, *P* = 0.25) (Fig. 2E and F).

### Relationship between predator and *S. nigricans* density

**Figure 3:**
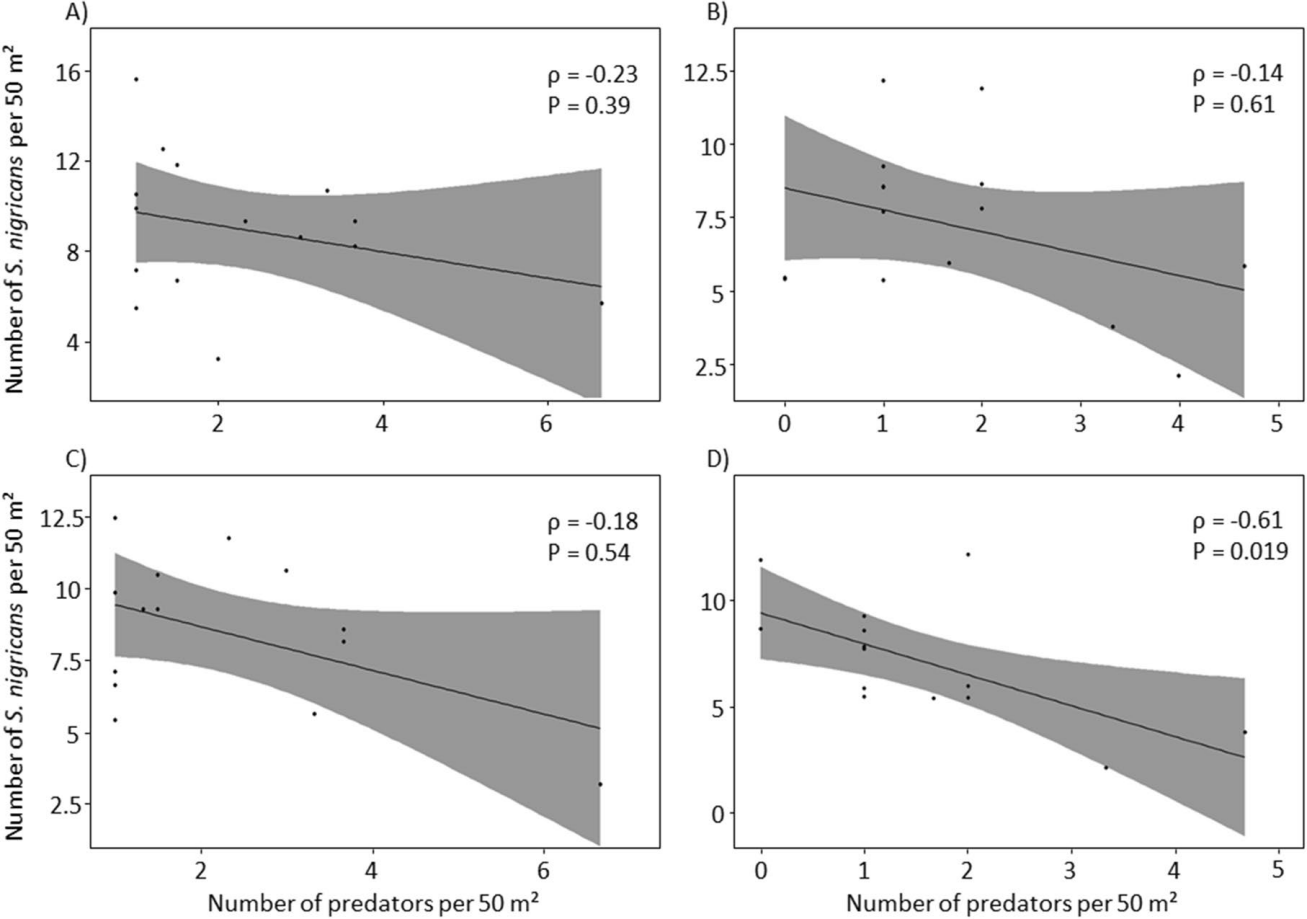
Correlation between the density of predators and the density of *Stegastes nigricans* in the year *n* on the reef flats inside (**A**) and outside (**B**) MPAs, and in the year *n* + *1* on the reef flats inside (**C**) and outside (**D**) MPAs. The grey areas represent the 95% confidence intervals.

Overall, the density of fish predators was not correlated with the density of damselfish within the same year inside (ρ = −0.23, S = 694, *P* = 0.39) or outside of the MPAs (ρ = −0.14, S = 640, *P* = 0.61) (Fig. 3A and B). However, there was a significant negative relationship between predator density and *S. nigricans* density in the following year outside of MPAs (ρ = −0.61, S = 734, *P* = 0.019) but not inside of MPAs (ρ = −0.18, S = 536, *P* = 0.54) (Fig. 3C and D). A correlation analysis performed for data from 2005 to 2010 (before predators density stabilized, see above) revealed that over that period, the same trend existed with the density of fish predators being negatively correlated with the density of *S. nigricans* in the following year inside the MPAs as well (ρ = −0.80, S = 61, *P* = 0.05).

### Relationship between competitors and *S. nigricans* density

The density of fish competitors was not correlated with the density of damselfish within the same year inside (ρ = 0.14, S = 482, *P* = 0.62) or outside of the MPAs (ρ = −0.04, S = 584, *P* = 0.88). The same was observed between competitor density and *S. nigricans* density in the following year (inside of MPAs: ρ = −0.13, S = 514, *P* = 0.66; outside of MPAs: ρ = −0.44, S = 654, *P* = 0.12).

### Relationship between habitat composition and *S. nigricans* density

The proportions of live coral, algal turf, and macroalgae were not correlated with the density of damselfish within the same year inside (live coral: ρ = −0.15, S = 646, *P* = 0.58; algal turf: ρ = 0.02, S = 547, *P* = 0.93; macroalgae: ρ = −0.13, S = 634, *P* = 0.64) or outside of the MPAs (live coral: R = 0.27, t = 1.00, *P* = 0.33; algal turf: R = 0.38, t = 1.48, *P* = 0.16; macroalgae: R = 0.25, t = 0.93, *P* = 0.37). The same was observed for live coral, algal turf, macroalgae and *S. nigricans* density in the following year (live coral: ρ = −0.17, S = 534, *P* = 0.41; algal turf: R = −0.23, t = −0.84, *P* = 0.42; macroalgae: ρ = −0.17, S = 535, *P* = 0.55 for inside the MPAs ; live coral: R = −0.20, t = −0.72, *P* = 0.48; algal turf: R = 0.04, t = 0.13, *P* = 0.90; macroalgae: R = −0.09, t = −0.30, *P* = 0.77 for outside the MPAs).

## Discussion

Our long-term monitoring data add to the ongoing debate regarding the role that *Stegastes* play as macro-scale ecosystem engineers on coral reefs, and provides insights into the factors that regulate their abundances. Over the 15 year study period (2005–2019) the density of *Stegastes* increased from 0.05 to 0.24 fish per m^2^. This increase was not associated with changes in the cover of live hard corals, macroalgae, or algal turf inside or outside the MPAs (Fig. 2). However, while we found no relationship between predator density and *S. nigricans* densities in the same year, a significant negative relationship was found across the whole study period outside of MPAs when considering predator density from the previous year, and this negative relationship was significant both inside and outside of MPAs during periods where predator density was relatively high (e.g. 2005–2010) (Fig. 3). These data suggest that predators may play a role in regulating the density of this species on coral reefs, especially when predator densities are high.

While correlative, our results provide insights into some of the ecological factors that may regulate the abundance of *S. nigricans*. Most notably, our data suggest that high predator densities may suppress the abundances of *S. nigricans* in the following year. This result was expected and is consistent with the findings of several comparable studies^29,34^; however, the density of predators was only high for part of our study period (2005–2011). Predator densities were consistently low between the years 2012–2015, after which it rose again slightly towards the final years of the study. While it is difficult to discern whether predator densities were unusually low between 2012 and 2015, or whether they were unusually high between 2005 and 2011, it may be worth noting that Moorea Island was hit by Cyclone Oli in 2010 and the observed decrease in predator abundance appears to align with that. Furthermore, while predator abundances decreased and stabilized at low levels in the years following Cyclone Oli, the abundance of *S. nigricans* increased both within and outside of the MPA areas. In addition to predator densities being generally low during this period ^35^, the increase in abundance of *S. nigricans* is also likely a product of increased coral damage by the cyclone, which can support the development of *Stegastes*’ farms^25^.

In contrast to past studies^19,36,37^, we observed no significant relationships between habitat substrates (hard corals, algal turf and macroalgae) and *S. nigricans* abundances. Interestingly, as similar to our finding that only high predation density affected *S. nigricans* density, the lack of an effect of *S. nigricans* on substrate composition may be due to their generally low densities around Moorea Island. For instance, we found that *S. nigricans* abundance increased to 0.24 fish per m^2^ over the course of this study, which is substantially less than the 4.2 fish per m^2^ reported by the study by Wilkes et al.^37^ in Florida, which found that *Stegastes* may have substantial effects on benthic community dynamics. Additionally, Ceccarelli et al.^18^ reviewed the role of territorial damselfishes as determinants of benthic communities, and concluded that most observations and experiments have been undertaken at the scale of individual territories, and found a strong influence of *Stegastes* on benthic communities^24,26,37,38^. As our work is a long-term study over a large geographic area, it differs from most of these past studies. We are cautious about drawing major conclusions on the role that *S. nigricans* play as a habitat modifier, given our results and their relatively low density around Moorea Island.

Overall, our long-term study conducted over a large spatial scale suggests that the density of *S. nigricans* around Moorea Island was not associated with variations in substrate composition or the density of their competitors. Instead, we found a negatively association with the density of their predators, but only when predator densities were relatively high. While correlative, our results complement the results of past work on this topic, which have tended to be much more targeted in their investigations. As a correlative study, we were unable to investigate the direct/indirect effects of additional factors such as the bleaching events that occurred in 2016 and 2019, ocean acidification, or temperature rise. Nonetheless, our results would suggest that predators may play a role in regulating the populations of keystone species, such as algae-farming damselfishes, in order to avoid “algal phase-shifts” in coral reef ecosystem.

## Methods

### Sampling sites

In 2004, the Fisheries Service of French Polynesia and the CRIOBE a monitoring program surveying eight Marine Protected Area sites (MPA—no-take zones) and five non Marine Protected Area sites (non-MPA—no fishing restriction) was set up around Moorea Island, French Polynesia^33^. A total of 13 sites were selected to monitor coral reef biodiversity around Moorea: eight Marine Protected Areas (MPA sites) and five non-Marine Protected Areas (non-MPA sites). Each MPA or non-MPA site extends from the shore to beyond the reef crest and out to the 70 m isobath on the outer reef slope. Three distinct reef habitats are thus surveyed at each site: the shallow reef flat (or fringing reef), the barrier reef, and the outer slope. On the shallow reef flats of the 13 sites, three permanent 25 m linear transects placed at the center of a 2 m-wide belt were located using a handheld global positioning system (GPS) receiver and some stakes fixed onto the reef. Since 2004, the 13 shallow reef flat sites had the same disturbance histories: a crown-of-thorns starfish (COTS) *Acanthaster* cf. *solaris* outbreak from 2006 to 2009, a cyclone in 2010, and weak bleaching events in 2016 and 2019. *Stegastes nigricans* are mainly found on shallow reef flats (i.e., reefs contiguous to the coast—less than 400 m from the coastline) around Moorea^32,39–41^. Therefore, within each site (eight MPA and five non-MPA sites), only shallow reef flats were considered this study (Fig. 1). The surveys were done between 8:00 and 11:00am, once per year (in February—warm and wet season).

### Fish surveys

For the fish surveys, a belt transect with an underwater visual censuses sampling technique (snorkeling) was used. A 25 m linear transect was placed at the center of a 2 m-wide belt. All fish seen along the transect were identified to the species level. On the first transect pass, the observer recorded highly mobile fish that entered the transect but usually fled as a snorkeler approached. On the second pass, less mobile, cryptic, and site-attached species were targeted with more detailed examinations of crevices. For substrate surveys, the cover proportions of live hard corals, dead corals with algal turf, macroalgae, sand, rubble, and others (e.g. anemones, shells, soft corals) were sampled using the Point Intercept Transect method every 50 cm over 25 m of the belt transect used for the fish survey. Sampling was conducted along three transects (three replicates) separated by 25 m at each site. The three belt transects were set up in the middle of each shallow reef flat (depth: 1 m).

A total of 221 fish species were identified on the fringing reef of all MPA or non-MPA sites at Moorea^35^. The classification of these species as predators or competitors of *S. nigricans* was based on direct observations made during previous research at these locations^11,39–43^. When research on a particular species was not available in the literature, we relied on expertise provided by Prof. Galzin, who has worked on coral reef fishes at these locations continuously since 1974^39–43^. Among the 221 recorded species, those within the families Serranidae (*Epinephelus hexagonatus, E. merra* and *Cephalopholis argus*), Muraenidae (*Gymnothorax javanicus, G. meleagris, G. undulates* and *Echidna nebulosa*) and Scorpaenidae (*Scorpaenopsis diabolus, Pterois antennata* and *P. radiata*) were considered to be predators of *S. nigricans*. Likewise, the species identified as competitors of *S. nigricans* (i.e., able to eat the algal turf farmed by *S. nigricans*) were *Ctenochaetus striatus, Acanthurus nigrofuscus, A. triostegus* and some Scaridae species (*Chlorurus spilurus, Scarus frenatus, S. globiceps, S. oviceps, S. rubroviolaceus, and S. schlegeli*). Moreover, some other territorial damselfish such as *Chromis viridis, Dascyllus aruanus,* and *Pomacentrus pavo* may compete with *S. nigricans* for space. As only adult fish are likely to be competitors/predators of *S. nigricans*, we only considered *S. nigricans*, predators, and competitors that reached their adult sizes in our analyses^44^.

### Statistical analyses

The normality of the distributions of the density of *S. nigricans,* predators, and competitors was tested with Shapiro–Wilk tests (W = 0.53 – 0.61; *P* < 10^−3^), like the distributions of the proportion of hard live corals, algal turf, and macroalgae (W = 0.64 – 0.87; *P* < 10^−3^). Temporal trends for these variables were then evaluated from 2005 to 2019 both inside and outside MPAs with modified Mann–Kendall tests for serially correlated data using the approach proposed by Hamed and Rao^45^ with variance correction to address potential autocorrelation, using the R package "modifiedmk”^46^.

The annual average density of *S. nigricans* was correlated to the annual density of its predators and competitors with Spearman’s correlations tests. The annual average density of *S. nigricans* was correlated to the annual proportions of live coral, algal turf, and macroalgae with Spearman’s or Pearson’s correlations tests. As a latency in the response may occur, the annual average density of *S. nigricans* in year *n* + *1* was also correlated to the annual density of predators, competitors and the proportions of live coral, algal turf, and macroalgae in year *n*. In our survey protocol, *S. nigricans* juveniles become adult fish after one year on the reef^40^. Moreover, predators (Serranidae, Muraenidae and Scorpaenidae) may eat juvenile *S. nigrican*s^32^. Therefore, we correlated the annual average density of *S. nigricans* in year n and n + 1 to the annual density of predators in year n. The statistical analysis was conducted using R-Studio and R version 3.5.1^47,48^ at the significance level α = 0.05.

### Ethical approval

This study did not involve endangered or protected species and was carried out in accordance with the guidelines of the French Polynesia Code de l’Environnement for animal ethics and scientific research (https://www.service-public.pf/diren/partager/code/). Moreover, the visual surveys (no experiment conducted on fish) were approved by SNO CORAIL (licensing committee: PGEM 2004—http://observatoire.criobe.pf/wiki/tiki-index.php). Lastly, the study was carried out in compliance with the ARRIVE guidelines (http://www.nc3rs.org.uk/page.asp?id=1357) to improve the reporting of research involving animals.

## Data Availability

All data generated and analysed during this study are available upon reasonable request to the corresponding author (DL).
